# Design of a Multi-Epitope Vaccine against *Tropheryma whipplei* Using Immunoinformatics and Molecular Dynamics Simulation Techniques

**DOI:** 10.3390/vaccines10050691

**Published:** 2022-04-28

**Authors:** Thamer H. Albekairi, Abdulrahman Alshammari, Metab Alharbi, Amal F. Alshammary, Muhammad Tahir ul Qamar, Tasneem Anwar, Saba Ismail, Bilal Shaker, Sajjad Ahmad

**Affiliations:** 1 Department of Pharmacology and Toxicology, College of Pharmacy, King Saud University, Riyadh 11451, Saudi Arabia; thalbekairi@ksu.edu.sa (T.H.A.); abdalshammari@ksu.edu.sa (A.A.); mesalharbi@ksu.edu.sa (M.A.); 2 Department of Clinical Laboratory Sciences, College of Applied Medical Sciences, King Saud University, Riyadh 11433, Saudi Arabia; aalshammary@ksu.edu.sa; 3 Department of Bioinformatics and Biotechnology, Government College University Faisalabad, Faisalabad 38000, Pakistan; 4 Department of Biosciences, COMSAT University, Islamabad 45550, Pakistan; tasneemanwar955@gmail.com; 5 Department of Biological Sciences, National University of Medical Sciences, Rawalpindi 46000, Pakistan; sabaismail7@gmail.com; 6 Department of Biomedical Engineering, Chung-Ang University, 84 Heukseok-ro, Dongjak-gu, Seoul 06974, Korea; ch.bilal321@outlook.com; 7 Department of Health and Biological Sciences, Abasyn University, Peshawar 25000, Pakistan

**Keywords:** *Tropheryma whipplei*, biophysical approaches, immunoinformatic, TLR-4

## Abstract

Whipple’s disease is caused by *T. whipplei*, a Gram-positive pathogenic bacterium. It is considered a persistent infection affecting various organs, more likely to infect males. There is currently no licensed vaccination available for Whipple’s disease; thus, the development of a chimeric peptide-based vaccine against *T. whipplei* has the potential to be tremendously beneficial in preventing Whipple’s disease in the future. The present study aimed to apply modern computational approaches to generate a multi-epitope-based vaccine that expresses antigenic determinants prioritized from the core proteome of two *T. whipplei* whole proteomes. Using an integrated computational approach, four immunodominant epitopes were found from two extracellular proteins. Combined, these epitopes covered 89.03% of the global population. The shortlisted epitopes exhibited a strong binding affinity for the B- and T-cell reference set of alleles, high antigenicity score, nonallergenic nature, high solubility, nontoxicity, and excellent binders of DRB1*0101. Through the use of appropriate linkers and adjuvation with a suitable adjuvant molecule, the epitopes were designed into a chimeric vaccine. An adjuvant was linked to the connected epitopes to boost immunogenicity and efficiently engage both innate and adaptive immunity. The physiochemical properties of the vaccine were observed favorable, leading toward the 3D modeling of the construct. Furthermore, the vaccine’s binding confirmation to the TLR-4 critical innate immune receptor was also determined using molecular docking and molecular dynamics (MD) simulations, which shows that the vaccine has a strong binding affinity for TLR4 (−29.4452 kcal/mol in MM-GBSA and −42.3229 kcal/mol in MM-PBSA). Overall, the vaccine described here has a promising potential for eliciting protective and targeted immunogenicity, subject to further experimental testing.

## 1. Introduction

*Tropheryma whipplei* is a rod-shaped Gram-positive bacterium that belongs to the phylum Actinobacteria and the order Actinomycetales [1]. It is recognized as a causative agent of Whipple’s disease and endocarditis [2]. Whipple illness, which is extremely rare and persistent, is involved in affecting various human organs, including the brain, eyes, skin, joints, and heart [3]. A higher percentage of males are more likely to contract the infection. It has been reported that Whipple’s disease is an uncommon infectious ailment that primarily infects males (73–87%) and affects them at an early adulthood age of 48–54 years. Chronic diarrhea, other chronic gastrointestinal symptoms (72–81%), and weight loss (79–93%) are the most common symptoms reported by individuals affected by the disease. Arthritis is the most common symptom reported by these patients (73–80%) [4]. In addition to conventional gastrointestinal involvement, other acute and chronic [5] *T. whipplei* localized symptoms have been documented. About 15% of pathogen infections are without symptoms. It might be fatal if not treated properly, but the mortality rate is still unknown [6]. Humans are the only carriers of *T. whipplei,* and the pathogen is spread by the oral–oral and oral–fecal pathways. Once a patient has developed an immunological response to the first encounter, the bacterium can be carried by the patient for an extended period of time, allowing it to spread across the community. Two investigations have found that antibodies against *T. whipplei* are present in 48% and 72% of the general population in Europe and Senegal, respectively, according to the researchers [7]. Despite this, clinical signs of this pathogen have only been described in a few cases around the world. This Gram-positive bacterium is present in sewage, soil, and drinking water [8]. It was reported that 6–11% of stool samples of noninfected individuals contain *T. whipplei* [9]. One-third of the infected individuals are more likely to exhibit long-lasting neurological changes regardless of the treatment [10]. It is a common bacterium of the gut, as the bacterial load observed in the saliva sample was much higher than in the stool sample [11]. It replicates in macrophages of intestinal mucosa [12]. *T. whipplei*, being the only known pathogen with a reduced genome (927 kb), is an actinobacterium with a GC content of 46% [13]. It lacks the genes involved in amino acid synthesis and energy metabolism [14]. After being phagocytized, the pathogen can survive [15]. The bacterium shows less variation, and a 99% similarity was reported between different strains of *T. whipplei* [16].

No specific diagnostic procedures are available [17]. Known diagnostic methods include polymerase chain reaction (PCR), duodenal biopsy, serology, immunohistochemistry, and histopathology [18,19]. Immunosuppression plays a role in making the person vulnerable to contracting a chronic infection. Reduction in the B and T cells is common. IgG and IgM concentrations remain the same during the infection but there is an increased production of IgA [20] in untreated infected patients. According to an observation, macrophages with inactivated cytokines support the replication of the pathogen [21,22,23]. Immunological defects showed their correlation with the disease [23]. Regarding the epidemiological features of the infection, it was reported that Asia and Africa are endemic regions. Although rare, 12 cases per year have been observed globally [24].

It was observed that the pathogen may become more pathogenic in the future, as witnessed by a recent report suggesting severe pneumonia and diarrhea caused by infection with *T. whipplei* [25]. In addition, there is no proper availability of diagnostic and treatment options, and the increasing antibiotic-resistant mechanisms altogether warrant the development of a novel vaccine for the pathogen [25,26,27,28]. The utilization of the core proteome and the concept of reverse vaccinology can make it easier to obtain vaccine targets. Vaccines can provide long-lasting protection compared to antibiotics and is not associated with the evolution of new resistance [29]. Approaches based on immunoinformatics are leading to the idea of vaccine design with the perception of being effective, less time-consuming, and aiding in combating various pathogenic infections [30].

The goal of this work was to identify extracellular, periplasmic, and outer membrane proteins, which are surface proteins that were used in the development of the MEPTWV (Multi-epitopes peptide *T. whipplei* vaccine). Due to the reduced genome size of *T. whipplei*, only 11 extracellular proteins were analyzed, leading to epitope prioritization resulting in four epitopes for vaccine construct design. The calculations of physicochemical parameters and the modeling of the vaccine construct structure, optimization, and validation of models were all conducted. Molecular docking of the vaccine with Toll-like receptor 4 (TLR-4) followed by molecular dynamics simulations were used to understand the interaction mechanisms and binding energies of the designed vaccine. In addition, it was understood how the host immune system responded to the vaccine.

## 2. Material and Methods

**Figure 1:**
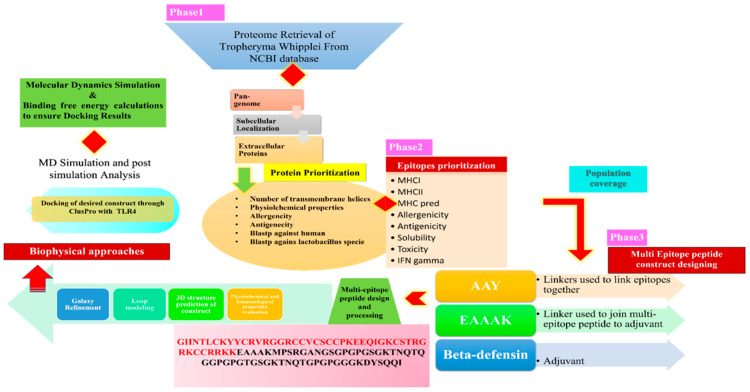
Schematic diagram highlighting all the steps in detail being used to proceed the study.

The whole in silico analysis utilized in this study to develop a multi-epitope vaccine against *T. whipplei* is depicted in Figure 1. 

### 2.1. Pan-Genome Analysis and Retrieval of Targeted Proteins

Complete sequenced proteomes of *T. whipplei* were retrieved from the NCBI [31] genome database and subjected to BPGA software [32] for core proteome extraction. This was followed by CD-HIT (cluster database at high identity with tolerance) analysis with a threshold of 90% to extract nonredundant sequences. All nonredundant sequences were subjected to PSORTB [33] to identify bacterial surface proteins. Only extracellular, periplasmic, and outer membrane sequences were collected for further analysis and MEPTWV design. As only extracellular proteins were obtained, they were examined further.

### 2.2. Choosing the Potential Vaccine Candidates

The sequences were evaluated against the presence of transmembrane helices less than 2 using TMHMM 2.0. The Protparam tool by expasy was used to evaluate molecular weight, therapeutical index, instability index, and GRAVY (to obtain hydrophilicity). Proteins sequences with molecular weights less than 100 kDa and instability indexes less than 45 (indicates a stable protein) were shortlisted and underwent further checks such as allergenicity and antigenicity using Allertop 2.0 [34] and Vaxijen 2.0 [35] with a threshold of 0.5, respectively. Proteins sequences resulting in allergens and nonantigens were discarded. Antigen proteins are considered important candidates for vaccines because these can interact with antibodies that result in humoral or cellular immune responses [36]. Then, BLASTp [37] was run against humans and lactobacillus species for these protein sequences, and those with a sequence identity greater than 30% were discarded.

### 2.3. Epitope Mapping

The shortlisted and final protein sequences meeting the criteria of being potential candidates for MEPTWV design went through the epitope mapping using the IEDB (Immune epitope analysis database) [38]. The B-cell epitope prediction tool of IEDB was used for B-cell epitope mapping where input sequences were entered in FASTA format. Only linear epitopes were used as they are capable of binding to antibodies even after denaturation. The predicted B-cell epitope was used for the prediction of MHC-II binding epitopes and then the predicted MHC-II binding epitopes were used as input sequences for the prediction of MHC-I binding epitopes. For T-cell epitopes (MHC-I and MHC-II binding), a complete reference set of alleles was selected. These predicted epitopes were analyzed and subjected to MHCPred [39], and epitopes with IC_50_ values less than 100 nM were selected and scrutinized further against allergenicity, antigenicity, toxicity, and solubility. The toxicity and solubility of epitopes were determined via ToxinPhred [40] and Protein-Sol [41], respectively. Only nonallergens, antigens, nontoxins, and soluble epitopes were selected for MEPTWV design. 

### 2.4. Population Coverage 

Population coverage for the shortlisted 4 epitopes was evaluated and the population coverage of IEDB was the tool used [42]. Population coverage analysis was performed using MHC Class I and II alleles covering the whole world, not only a specific region, aiming toward the strategy of designing a vaccine targeting the world population. The MHC class I and II binding allele set used for the analysis included HLA-A*01:01,HLA-A*01:01,HLA-A*02:01,HLA-A*02:01,HLA-A*02:03,HLA-A*02:03,HLA-A*02: 313206,HLA-A*02:06,HLA-A*03:01,HLA-A*03:01,HLA-A*11:01,HLA-A*11:01,HLA-A*23:01,HLA-A*23:01,HLA-A*24:02,HLA-A*24:02,HLA-A*26:01,HLA-A*26:01,HLA-A*30:01,HLA-A*30:01,HLA-A*30:02,HLA-A*30:02,HLA-A*31:01,HLA-A*31:01,HLA-A*32:01,HLA-A*32:01,HLA-A*33:01,HLA-A*33:01,HLA-A*68:01,HLA-A*68:01,HLA-A*68:02,HLA-A*68:02,HLA-B*07:02,HLA-B*07:02,HLA-B*08:01,HLA-B*08:01,HLA-B*15:01,HLA-B*15:01,HLA-B*35:01,HLA-B*35:01,HLA-B*40:01,HLA-B*40:01,HLA-B*44:02,HLA-B*44:02,HLA-B*44:03,HLA-B*44:03,HLA-B*51:01,HLA-B*51:01,HLA-B*53:01,HLA-B*53:01,HLA-B*57:01,HLA-B*57:01,HLA-B*58:01,HLA-B*58:01,HLA-DRB1*01:01,HLA-DRB1*03:01,HLA-DRB1*04:01,HLA-DRB1*04:05,HLA-DRB1*07:01,HLA-DRB1*08:02,HLA-DRB1*09:01,HLA-DRB1*11:01,HLA-DRB1*12:01,HLA-DRB1*13:02,HLA-DRB1*15:01,HLA-DRB3*01:01,HLA-DRB3*02:02,HLA-DRB4*01:01,HLA-DRB5*01:01,HLA-DQA1*05:01/DQB1*02:01,HLA-DQA1*05:01/DQB1*03:01,HLA-DQA1*03:01/DQB1*03:02,HLA-DQA1*04:01/DQB1*04:02,HLA-DQA1*01:01/DQB1*05:01,HLA-DQA1*01:02/DQB1*06:02,HLA-DPA1*02:01/DPB1*01:01,HLA-DPA1*01:03/DPB1*02:01,HLA-DPA1*01:03/DPB1*04:01,HLA-DPA1*03:01/DPB1*04:02,HLA-DPA1*02:01/DPB1*05:01,HLA-DPA1*02:01/DPB1*14:01.

### 2.5. Multi-Epitope Vaccine Design

The epitopes being potential vaccine candidates were linked together using GPGPG linkers [43]. The EAAAK linker was used to connect the epitope with the adjuvant usually used to boost up the action activity of vaccines [44]. The addition of adjuvant can enhance the overall immunogenicity of the multi-epitope peptide [45]. To construct the epitopes peptide, GPGPG linkers were used as they help facilitate epitopes presentation to the immune system and allow efficient immune processing. The EAAAK linkers were utilized to combine the first CTL epitope and adjuvant and to isolate the domains of a bi-functional fusion protein [46]. B-defensin was used as an adjuvant of this MEPTWV [47]. Biochemical properties such as molecular weight, hydrophilicity, and therapeutic and instability index of the vaccine construct were obtained by Protparam of the Expasy tool [48]. Scratch predictor [49] is a tool for 3D structure prediction, and the vaccine construct designed using epitopes was submitted to Scratch predictor for 3D structure prediction. Once we obtained the model of our vaccine construct, the procedure was followed by loop modeling and refinement by Galaxy loop and Galaxy refine of Galaxy Web [50].

### 2.6. Disulfide Engineering and In Silico Cloning

To attain structural stability, disulfide bonds were introduced in the designed MEPTWV, while the residue pairs were mutated to cysteine using the online server Disulfide by design [51]. Codon optimization of the vaccine construct was performed and evaluated based on the CAI value and GC content by Jcat (Java codon adaptation tool) [52]. Codon optimization was followed by in silico cloning by Snapgene [53], and the vaccine model (DNA sequence obtained from Jcat) was expressed in the pET-28a (+) expression vector.

### 2.7. Computational Immune Simulation

The C-ImmSim server [54] was employed for the prediction and analysis of immune–epitope interaction via machine learning techniques. MEPTWV was tested against the probability of being immunogenic and the ability to induce immunogenicity.

### 2.8. Molecular Docking

Molecular docking of the MEPTWV model was performed with TLR-4 (Toll-like receptor 4), which is involved in producing cytokines, leading to the activation of the innate immune response. Binding predictions of the vaccine with the human receptor were analyzed under this step. The ClusPro 2.0 [55] online tool was used for molecular docking.

### 2.9. Molecular Dynamics Simulation

The system dynamics in an aqueous solution was assessed using AMBER20 software. The padding distance of 12 angstroms was maintained between the protein and water box edges. The ff14SB force field was employed to set the parameters of both the vaccine and the TLR-4, leading to the inclusion of the complex into the TIP3P water box. The hydrogen atoms, nonheavy atoms, carbon alpha atoms, and solvation box were reduced to 500, 300, 1000, and 1000 steps, respectively, when Na+ ions were injected to neutralize the system. Langevin dynamics performed system heating to 300 K for 20 ps for system temperature maintenance. It was achieved using a time step of 2 fs and a detachment of 5 kcal/mol-A2 on the carbon alpha atom. For stability, the system was slowed to 100 frames per second. The NPT ensemble assisted in maintaining the system’s pressure for 50 ps, allowing the production run of 150 ns for 2 fs to be completed. The trajectories acquired were analyzed using AMBER CPPTRAJ [56].

### 2.10. MM-PB/GBSA Studies

The MMPBSA.py package of the AMBER18 program was involved in calculating the intermolecular binding free energies [57]. Evaluation of the free energy difference in solvated and gas phases of the complex was carried out.

Evaluation of the net binding free energy:

ΔGbindingfreeenergy = ΔGbind,vaccum + ΔGsolv,complex − (ΔGsolv,ligand) + ΔGsolv,receptor

ΔGsolv = ΔGelectrostatic, ε = 80 + ΔGelectrostatic, ε = 1 + ΔGhydrophobic

ΔGvaccum = ΔEmolecular,mechanics − T⋅ΔGnormalmodeanalysis

## 3. Results

### 3.1. Core Proteome Retrieval

Two complete proteomes of *T. whipplei* were retrieved from NCBI and used for pan-genome analysis using BPGA [58]. Core proteomes were obtained and used as vaccine targets against the pathogen. They were subjected to CD-HIT with a threshold of 90%, resulting in the removal of duplicate sequences, leaving behind only nonredundant sequences, which were 802. Only 11 extracellular protein sequences had shown their presence and were extracted from the data obtained from PsortB and shortlisted for further analysis.

### 3.2. Determination of Potential Vaccine Candidates

The core surface potential vaccine candidates were used in reverse vaccinology (RV) to obtain potential vaccine candidates [59]. The extracellular proteins were analyzed for the presence of transmembrane helices, and those with less than or equal to 1 were considered acceptable. Out of 11 core proteins, only one had 2 transmembrane helices, which were discarded because proteins having more than 2 transmembrane helices are not regarded as good candidates [60]. The remainder with no transmembrane helices were evaluated for molecular weight, GRAVY, therapeutic, and instability index. Molecular weight is supposed to be less than 100 kDa, as proteins with a molecular weight of less than 100 are easier to purify [61]. All proteins except those showing the presence of transmembrane helices and having a molecular weight less than 100 kDa were considered further. Three core proteins were unstable, having an instability index more than 45, while 7 proteins (core/531/1/Org1_Gene461 Length: 202, core/614/1/Org1_Gene722 Length: 159, core/655/1/Org1_Gene691 Length: 140, core/690/1/Org1_Gene723 Length: 120, core/711/1/Org1_Gene771 Length: 105, core/716/1/Org1_Gene749 Length: 103, core/738/1/Org1_Gene751 Length: 87, core/796/1/Org1_Gene758 Length: 49) were observed stable with instability indexes less than 45. Hydrophilicity was checked and the negative GRAVY value depicted nonpolar proteins. The proteins: core/531/1/Org1_Gene461 Length: 202, core/655/1/Org1_Gene691 Length: 140, core/711/1/Org1_Gene771 Length: 105, and core/796/1/Org1_Gene758 Length: 49 were nonallergens and then subjected to an antigenicity check. The proteins: core/655/1/Org1_Gene691 Length: 140, core/711/1/Org1_Gene771 Length: 105, and core/796/1/Org1_Gene758 Length: 49 were antigenic with antigenicity values of 0.6879, 0.7479, and 0.5907, respectively. These core proteins met the criteria of potential vaccine candidates and no significant similarity hit with human and lactobacillus species. The selected proteins as potential vaccine targets are tabulated in Table 1.

### 3.3. Epitope Prediction and Prioritization Leading to Vaccine Construct Formation

Epitopes were predicted by IEDB. B-cell epitopes prediction was performed using the B-cell epitope prediction of IEDB, resulting in 13 predicted peptides having the potential to be B-cell epitopes. B-cell epitopes binding to antibodies result in activating humoral immune responses. Screening was performed of the predicted peptides acting as B-cell epitopes for the T-cell epitopes that were supposed to have the binding sites for MHC class I and class II [60]. The predicted peptides having a length of less than 11 amino acid residues were discarded, and the remaining 9 peptides were submitted to predict MHC class II epitopes. A set of 7 reference alleles were used for the MHC class II epitopes prediction, while 16 MHC class II epitopes were predicted from the B-cell epitope based on their percentile ranks. The lower the percentile rank, the better the binders. Then, these 16 MHC Class II epitopes were used to derive MHC class I epitopes. In addition, 21 peptides acting as B-cell-derived T-cell epitopes were obtained. All those epitopes were analyzed under MHC pred to screen epitopes tending to bind with the allele DRB 0101, as this is the most widely distributed allele in the human population [60]. Epitopes showing strong binding interactions with the DRB 0101 allele result in strong immunological responses, and the epitopes having IC_50_ values less than 100 were picked for epitope prioritization to design vaccine constructs. Epitopes with a lower IC_50_ value <100 for T cell alleles were considered as high-affinity binders based on a competitive binding assay. In addition, 16 out of 25 with an IC_50_ value less than 100 were examined for allergenicity by AllerTop v.2.0. Five epitopes were observed as allergens, while the antigenicity check was applied on the remaining 11 nonallergen epitopes using vaxijen, in order to re-assure that the epitopes are strong enough to induce immunogenic responses. The threshold for being an antigenic epitope was set at 0.5, resulting in only 6 antigenic epitopes, followed by a toxicity check to remove toxic epitopes. Whether all of them were nontoxins were inquired for the solubility test. Finally, only 4 soluble epitopes including MPSRGANGS, SGKTNQTQG, TGSGKTNQT, and GGKDYSQQI were considered for vaccine design (Table 2).

Shortlisted epitopes meeting the criteria of being potentially immunological active and safe were linked together using GPGPG linkers. These linkers are rigid and allow efficient separation of epitopes. B-defensin was used as an adjuvant and was linked with epitopes by the EAAAK linker. The EAAAK linker keeps the linked epitopes peptide and adjuvant separated and does not allow their folding on one another. The adjuvant is usually used to improve the immune stimulation of the vaccine to provoke better immune responses including the induction of chemokines, cytokines, and presentation of antigens [61]. The designed construct for the multi-epitope vaccine was inspected for antigenicity and allergenicity, revealing to be antigenic with an antigenicity of 1.5203 and nonallergenic with a molecular weight of 10.326 kDa, and a GRAVY equal to −0.948 is the indication of being hydrophilic. The therapeutic index was 9.73, declaring the range at which the vaccine was believed as safe. In addition, the vaccine construct was observed stable with a stability index of 28.78.

### 3.4. Structure Modeling

**Figure 2:**
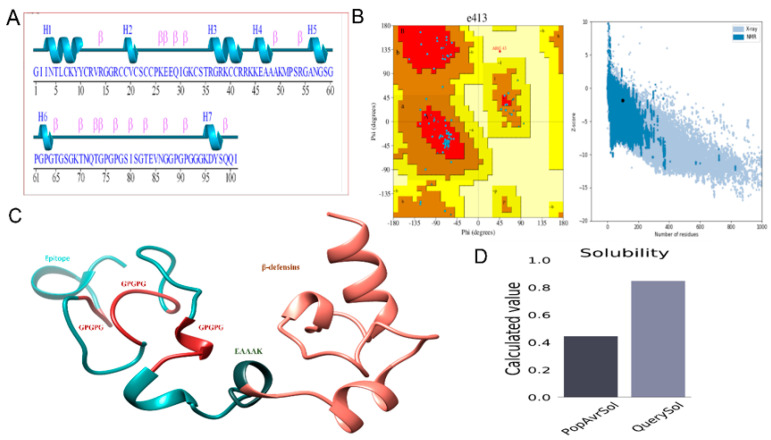
(**A**) Secondary structure for MEPTWV predicted through PDBSum. (**B**) Ramachandran plot depicting the validation of the structure along with the Graph presenting Z-score. (**C**) 3D model for multi-epitope peptide vaccine predicted by Scratch predictor. (**D**) Solubility graph.

A 3D model of the MEPTWV obtained by 3DPro of SCRATCH predictor and visualized by UCSF Chimera was submitted to the Pdbsum generator to obtain the secondary structure (Figure 2A). The structure showed alpha-helices at 16.83%, beta bridged at 0%, 3_10_ helixes at 0%, extended strands at 15.84%, Beta turns at 3.96%, and random coils at 63.37%. The Ramachandran plot and G-factor were also generated by Pdbsum (Figure 2B). Here, 59 residues were in the Rama-favored region, 10 in the additional allowed regions, and 1 in the disallowed region. There was the indication of 22 Glycine and 8 proline residues in the Ramachandran plot. The average score calculated for the G-factor was −0.05. Figure 2D represents the solubility score that was 0.8, while the average score should be 0.4. The detection of 2 loops: ILE2-TYR10 and SER34-ARG43 in the 3D model of MEPTWV, allowed loop modeling. The next step was refinement of the model that was executed by Galaxy refine of Galaxy web. Loop modeling is necessary for the structural conformation stability in loop regions [62]. Refinement of the model helps in improving the quality of the structure [63]. Model 1 was selected based on good Rama-favored regions, poor rotamers, and a molprobity score. The top model with structural information is given in Table 3. The two-dimensional structure of the vaccine construct along with the validation is also given [64].

### 3.5. Population Coverage

**Figure 3:**
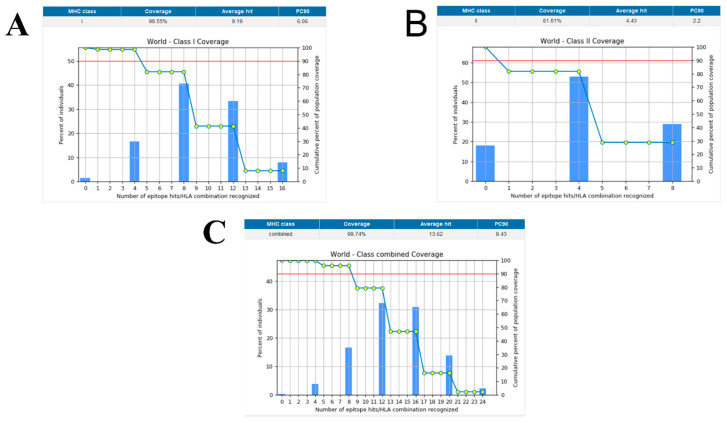
(**A**) World population coverage for MHC class I. (**B**) World population coverage for MHC Class II. (**C**) Combined world population coverage for HLA allele recognized as T-cell epitopes.

**Figure 4:**
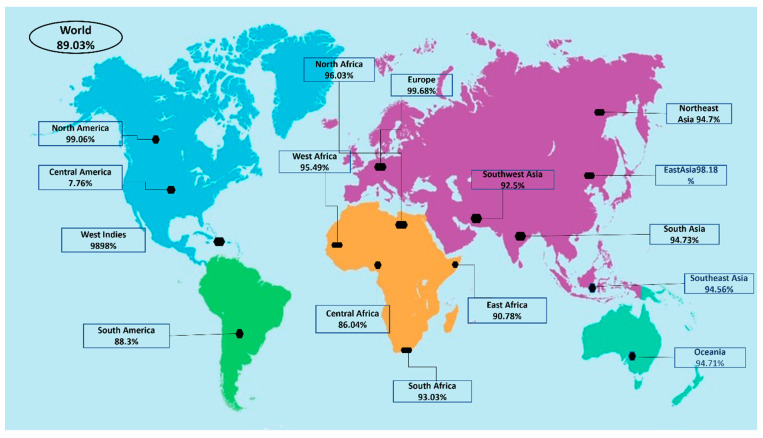
Region-wise world population coverage by vaccine epitopes.

Population coverage was analyzed by IEDB. MHC class I and II world population coverage was 98.55 and 81.81, respectively, against the epitopes considered as potential candidates for the vaccine to be designed, as graphically represented in Figure 3A,B. The world class-combined population is depicted in Figure 3C. Population coverage refers to the probability of epitopes binding to MHC molecules covering different regions of the world [65]. Figure 4 highlights the region-wise population coverage against the epitopes supposed to be part of the vaccine.

### 3.6. Disulfide Engineering and In Silico Cloning

**Figure 5:**
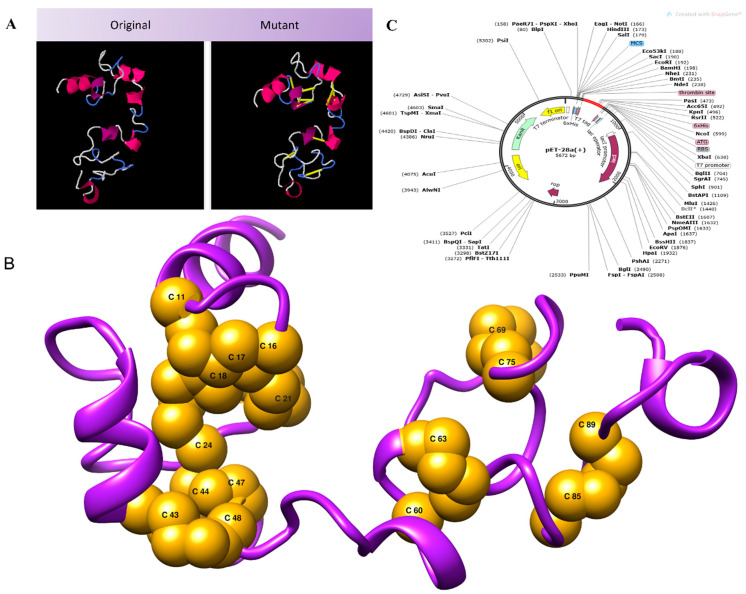
(**A**) Vaccine model along with the mutant model of vaccine. (**B**) 3D structure of the mutant model in which all the residues mutated to cysteine, introducing disulfide bonds. (**C**) Vaccine sequence expressed in pET28a (+) vector.

Disulfide engineering was performed for structural stability. It involved the introduction of disulfide bonds as cysteine pairs [66]. Nine residue pairs were spotted to have a high energy value and confer instability to the vaccine conformation. The residue pairs chosen to be mutated as a cysteine residue were 11CYS-16GLY(*X*3 angle, +72.29, energy value, 2.45 kcal/mol), 17ARG-21CYS(*X*3 angle, −75.15, energy value, 5.11 kcal/mol), 18CYS-24CYS(*X*3 angle, +113.81, energy value, 2.29 kcal/mol), 43ARG-48ALA(*X*3 angle, −75.78, energy value, 6.34 kcal/mol), 44LYS-47ALA(*X*3 angle, +90.53, energy value, 4.09 kcal/mol), 60GLY-63PRO(*X*3 angle, −115.50, energy value, 7.58 kcal/mol), 69LYS-75PRO(*X*3 angle, +90.13, energy value, 4.06 kcal/mol), 77PRO-85VAL(*X*3 angle, +89.94, energy value, 5.00 kcal/mol), and 89PRO-97TYR(*X*3 angle, +115.48, energy value, 5.39 kcal/mol). The wild and mutated vaccine structure is provided in Figure 5A. Figure 5B depicts the 3D model of the mutant vaccine, in which all mutated residues are shown. For in silico cloning, the vaccine sequence was reverse-translated. The CAI calculated was 1.0 with a GC content of 54.78. In silico cloning aided in determining the expression of the designed vaccine in the *E. coli* K12 strain. The DNA sequence of the vaccine construct being cloned into the vector was GGTATCATCAACACCCTGTGCAAATACTACTGCCGTGTTCGTGGTGGTCGTTGCTGCGTGCTCTTGCTGCCCGAAAGAAGAACAGATCGGTAAATGCTCTACCCGTGGTCGTAAATGCTGCCGTCGTAAAAAAGAAGCTGCTGCTAAAATGCCGTCTCGTGGTGCTAACGGTTCTGGTCCGGGTCCGGGTTCTGGTAAAACCAACCAGACCCAGGGTGGTCCGGGTCCGGGTACCGGTTCTGGTAAAACCAACCAGACCGGTCCGGGTCCGGGTGGTGGTAAAGACTACTCTCAGCAGATC. Figure 5C shows the sequence cloned into the vector using Snapgene. It was observed that disulfide engineering had an enhanced vaccine structural stability and removed degradation-prone regions of the vaccine.

### 3.7. Computational Immune Simulation

**Figure 6:**
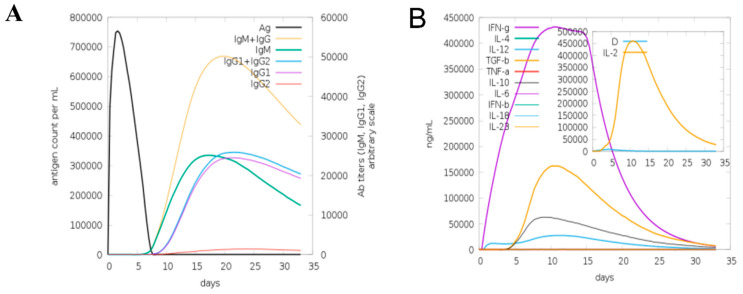
Computational immune simulation of host immune system against designed vaccine. (**A**) Computational immune simulation graph showing immunoglobulins production. (**B**) Graph showing the induction of cytokines and interleukins.

A computational immune simulation was performed to observe in silico immune responses against the designed vaccine. The results showed that it was proven effective in inducing immune responses as the production of immunoglobins was seen. Induction of interleukins and IFN-g (a cytokine) was also observed. Figure 6A is a graphical representation of computational immune responses against the vaccine. With the increasing number of days, the antibodies production was amplified from primary to secondary. Figure 6B depicts that IFN-g was produced on a higher scale, playing a vital role in pathogen clearance [67].

### 3.8. Molecular Docking of MEPV

**Figure 7:**
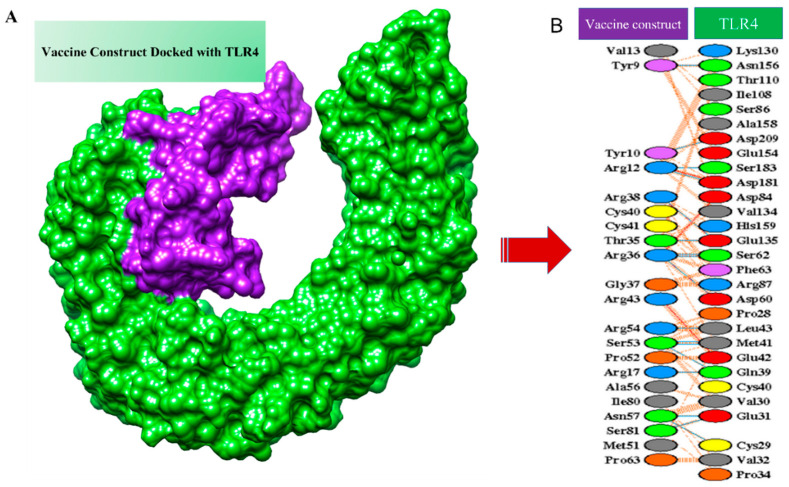
Docked complex. (**A**) Docked complex of designed vaccine with TLR4. (**B**) Protein–protein interaction of the vaccine and the receptor, which is TLR4.

TLR-4 was used as a receptor in molecular docking with the designed vaccine. TLR-4 is a Toll-like receptor involved in activating immunity and mediating cytokine induction [68]. Docking is an approach to predict the interaction of receptors and ligands (vaccine) [69]. Models obtained by docking were ranked based on clusters and different coefficients showing different energies. The first model was selected as it has the lowest binding energy. IRMSD was calculated for the pairs among the structure, leading to the selection of the structure with the highest number of neighbors within a 9 Å IRMSD radius. The structure selected, termed the center of the first cluster and structure present within the 9 Å IRMSD neighborhood of the center, will be the first cluster [70]. The score of the top models is listed in Table 4. The protein–protein interaction of the vaccine and TLR-4 is shown in Figure 7. Salt bridges are shown in red, disulfide bonds are shown in yellow, hydrogen bonds are shown in blue, and nonbonded interactions are shown in orange.

### 3.9. Molecular Dynamics Simulation (MD Simulation)

**Figure 8:**
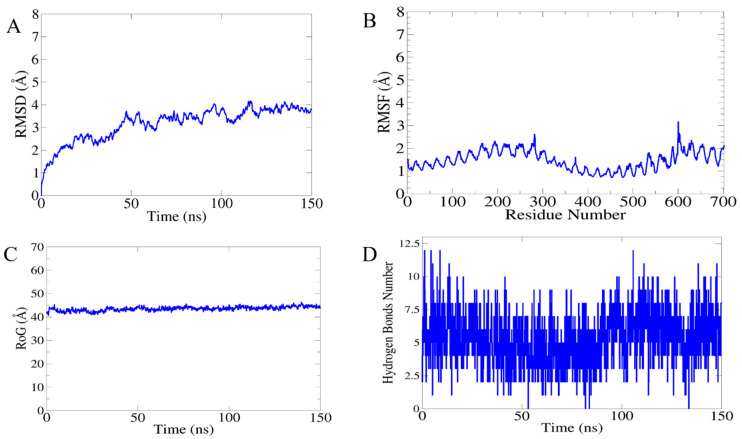
Statistical analysis of simulation trajectories: (**A**) root-mean-square deviation (RMSD), (**B**) radius of gyration (Rog), (**C**) root-mean-square fluctuation (RMSF), and (**D**) hydrogen bonding (H.B).

The molecular dynamics and stability of the docked complexes were explored through a 150 nanosecond (ns) run of a MD simulation. The resulting trajectories were evaluated through four different parameters: (i) root-mean-square deviations (RMSDs), (ii) root-mean-square fluctuation, (iii) radius of gyration (*Rg*) and hydrogen bonding (H.B), as mentioned in following Figure 8. The RMSD was calculated for the docked complex to check structure stability among the superimposed snap generated via simulation. The mean RMSD observed for the system was 4.9 Å (maximum of 4.95 Å observed at frame 120 nanoseconds, as shown in Figure 8A, while generally, the RMSD graph showed little changes, becoming stable at the end, and no drastic changes were observed. Next, the radius of gyration (*Rg*) calculation was performed, which was mainly estimated for describing the relaxation and compactness of proteins. The *Rg* estimated for the system was 40.47 Å (maximum level of variation of 42.42 Å). As mentioned in Figure 8B, the (*Rg*) results revealed that there was no higher variation in the plot, so the *Rg* plot predicted the compact behavior of TLR-4 in the presence of a vaccine molecule. Additionally, the residue-wise fluctuation was analyzed from the RMSF calculation, in which the mean residue fluctuation was 2.9 Å (maximum of 3.6 Å), as mentioned in Figure 8C. Moreover, the intermolecular interactions strength was evaluated through hydrogen bonding analysis that demonstrated the maximum formation of 11 hydrogen bonds in each frame of the simulation, as shown in Figure 8D.

### 3.10. Binding Free Energies

Binding free energies were predicted by both the molecular mechanics generalized Born surface area (MM-GBSA) and molecular mechanics Poisson–Boltzmann surface area (MM-PBSA), which are powerful methods to determine intermolecular stability. As can be seen in Table 5, the vaccine–receptor complex accomplishes a highly favorable net binding free energy of −29.4452 kcal/mol in MM-GBSA and −42.3229 kcal/mol in MM-PBSA. It was observed that the van der Waals energy dominated the system stability and played a favorable contribution to the net binding energy.

## 4. Discussion

Whipple’s disease is considered a rare type of infection as the incidence of the infection is low [71]. It was reported that people with some sort of genetic abnormalities in the immune system have a higher chance to contracting an infection [72]. The disease is currently treated with antibiotics, which are given to patients for 1–2 years. Some of the infections last less than a year. Some of the infected individuals show a response to the antibiotic [73]. Vaccination is the best way to combat infections compared to antibiotics as the latter pushes the bacteria to evolve new resistance mechanisms [74].

Herein, a multi-epitope peptide vaccine was designed against *T. whipplei*. Reverse vaccinology was applied to design an effective vaccine [75]. Targets were identified that could be part of the pathogen core proteome and nonredundant. Extracellular proteins being surface proteins were used and subjected to different vaccine filters to obtain the potential targets [76]. Proteins were examined for the presence of transmembrane helices, allergenicity, antigenicity, and physicochemical properties to screen experimental, feasible, and effective vaccine targets [77].

Epitope mapping of the selected core proteins was performed via immunoinformatic tools/server, and ten led to epitope prioritization [76]. B-cell-derived T-cell epitopes were predicted and selected on the basis of their antigenicity, nonallergenicity, solubility, and nontoxicity potential to be a part of the MEPTWV. The development of the MEPTWV was supposed to induce adaptive immunity, and it was also with better physicochemical properties in terms of stability, feasibility, and hydrophilicity. A high-quality 3D model of the vaccine construct was generated, with the majority of the amino acid residues located in the favored region of the Ramachandran plot, as determined by the Ramachandran plot. The MEPTWV must have a high affinity for the immunological receptor to be able to simulate both innate and adaptive immunity. The vaccine also had a high MHC molecule binding potential. Initial immune responses are triggered by these interactions, which are followed by adaptive immune responses in response to the epitope antigens that are exposed to the host immune system. In addition, molecular docking, MD simulation, and binding free energies [78] indicated that MEPTWV-TLR4 interactions were stable. A substantial number of intermolecular H-bonds were identified [79]. As a result of these findings, MEPTWV appeared to have a high affinity for immunological receptors. In silico cloning was utilized to confirm that the vaccine designs could be efficiently expressed in the pET-28a (+expression vector) vector [62,63,64,65,80]. Though the results of this study are promising [81], one limitation of the current work is the lack of experimental validation. In vivo and in vitro studies are needed to determine the MEPTWV’s anti-*T. whipplei* efficacy.

## 5. Conclusions

The use of pan-genomics, immunoinformatics, and subtractive proteomics to develop a safe and efficacious multi-epitope vaccine against *T. whipplei* infections might be an effective therapeutic and prophylactic option in the future. The study predicted several subunit and B and T-cell epitopes that can be used in a recombinant vaccine design against the pathogen. Linkers were used to connect the predicted epitopes. The vaccine design had a high affinity for the innate immune receptor (TLR-4), which aids in stimulating innate and adaptive immunity against pathogen infection. The simulations revealed highly stable molecular interactions and predicted stable intermolecular binding conformation. Furthermore, the binding free energy assessment of the complex showed that the complex was stable. Our in silico research revealed that the proposed vaccine was immunogenic, but the effectiveness of the vaccine in preventing *T. whipplei* infection needed to be tested. Such computer-aided vaccine strategies have been successfully applied in the past for vaccine development. Many pathogens genomes have been successfully screened for protective antigens, including the Meningococcal B (MenB) vaccine, which was approved by the Food and Drug Administration [38]. In addition to Chlamydia, Staphylococcus aureus, and group A *Streptococcus*, in silico techniques have been utilized to treat other infections [82].

## Figures and Tables

**Table 1:** Physiochemical properties of proteins shortlisted for vaccine design.

Core Protein Gene Ids	TMHMM	Molecular Weight	T. PI	Instability Index		Gravy	Allergenicity	Antigenicity	
core/655/1/Org1_Gene691 Length: 140	0	15.07	9.27	31.58	Stable	−0.539	Non-Allergen	0.6879	Selected
core/655/1/Org1_Gene691 Length: 140	0	15.07	9.27	31.58	Stable	−0.539	Non-Allergen	0.6879	Selected
core/796/1/Org1_Gene758 Length: 49	0	5.257	8.1	27.51	Stable	−0.678	Non-Allergen	0.5907	selected

**Table 2:** Epitope prioritizations using different filters to select epitopes having the potential to be part of the vaccine construct.

Proteins	B-Cell Epitopes	MHC-II	MHC I		MHC Pred	IC50 Value	Allergenicity	Antigenicity	Toxicity	Solubility
>core/711/1/Org1_Gene771	MPSRGANGSDTFLY	MPSRGANGSDT	MPSRGANGS	2.9	MPSRGANGS	22.18	Nonallergen	2.1437	NON-TOXIN	Soluble
	SNTWTYTGSGKTNQTQG	TGSGKTNQTQG	SGKTNQTQG	13	SGKTNQTQG	76.21	Nonallergen	2.7169	NON-TOXIN	Soluble
			TGSGKTNQTQ	12	TGSGKTNQT	58.88	Nonallergen	2.6625	NON-TOXIN	Soluble
>core/655/1/Org1_Gene691	DVLTKGGKDYSQQITT	KGGKDYSQQIT	GGKDYSQQI	5.1	GGKDYSQQI	53.09	Nonallergen	0.9704	NON-TOXIN	Soluble

**Table 3:** Refined models of the designed vaccine.

Model	Global Distance Test—High Accuracy (GDT-HA)	Root Mean Square Deviation (RMSD)	MolProbity	Clash Score	Poor Rotamers	Rama Favored
Initial	1	0	3.594	167.3	1.3	72.7
MODEL 1	0.9653	0.36	2.605	30	0	85.9
MODEL 2	0.9629	0.383	2.53	29.3	0	88.9
MODEL 3	0.9653	0.359	2.604	31.4	0	86.9
MODEL 4	0.9629	0.386	2.585	30	0	86.9
MODEL 5	0.953	0.409	2.582	31.4	0	87.9

**Table 4:** Top models of docked complexes of designed vaccine with TLR4.

Cluster	Members	Representative	Weighted Score
**0**	62	Center	−819.7
		Lowest Energy	−916.3
**1**	62	Center	−721.6
		Lowest Energy	−858.9
**2**	54	Center	−720.2
		Lowest Energy	−821.7
**3**	50	Center	−828.4
		Lowest Energy	−878
**4**	49	Center	−738.2
		Lowest Energy	−871.3
**5**	49	Center	−770.2
		Lowest Energy	−849.7
**6**	48	Center	−722.7
		Lowest Energy	−778.8
**7**	46	Center	−820.7
		Lowest Energy	−878.4
**8**	33	Center	−725.7
		Lowest Energy	−805
**9**	29	Center	−761.8
		Lowest Energy	−815.8
**10**	28	Center	−742.8

**Table 5:** Binding free energies in kcal/mol.

MM-GBSA				MM-PBSA			
Complex							
Energy Component	Average	Std. Dev.	Err. of Mean	Energy Component	Average	Std. Dev.	Err. of Mean
VDWAALS	−14,268.3	51.3606	5.1361	VDWAALS	−14,268.3	51.3606	5.1361
EEL	−125,777	160.9493	16.0949	EEL	−125,777	160.9493	16.0949
EGB	−23,027.7	128.2908	12.8291	EPB	−22,314.4	132.7034	13.2703
ESURF	616.7839	3.4972	0.3497	ENPOLAR	410.6266	1.8671	0.1867
G gas	−140,045	155.5427	15.5543	G gas	−140,045	155.5427	15.5543
G solv	−22,410.9	127.4705	12.7471	G solv	−21,903.8	132.0576	13.2058
TOTAL	−162,456	110.4932	11.0493	TOTAL	−161,949	124.2808	12.4281
Receptor:							
Energy Component	Average	Std. Dev.	Err. of Mean	Energy Component	Average	Std. Dev.	Err. of Mean
VDWAALS	−12,136.3	50.2011	5.0201	VDWAALS	−12,136.3	50.2011	5.0201
EEL	−10,4629	162.6708	16.2671	EEL	−104,629	162.6708	16.2671
EGB	−18,516.2	136.2444	13.6244	EPB	−17,941.6	136.4663	13.6466
ESURF	489.8555	3.2995	0.3299	ENPOLAR	330.3065	1.6312	0.1631
G gas	−116,765	155.9755	15.5976	G gas	−116,765	155.9755	15.5976
G solv	−18,026.3	135.2806	13.5281	G solv	−17,611.3	135.8915	13.5892
TOTAL	−134,791	95.4058	9.5406	TOTAL	−134,376	106.9958	10.6996
Ligand:							
Energy Component	Average	Std. Dev.	Err. of Mean	Energy Component	Average	Std. Dev.	Err. of Mean
VDWAALS	−1902.44	19.2818	1.9282	VDWAALS	−1902.44	19.2818	1.9282
EEL	−21,889.9	83.9904	8.399	EEL	−21,889.9	83.9904	8.399
EGB	−4010.19	70.0089	7.0009	EPB	−3857.43	65.2142	6.5214
ESURF	156.9794	1.8144	0.1814	ENPOLAR	109.2052	1.3425	0.1342
G gas	−23,792.4	88.4392	8.8439	G gas	−23,792.4	88.4392	8.8439
G solv	−3853.21	69.1563	6.9156	G solv	−3748.22	64.88	6.488
TOTAL	−27,645.6	41.7466	4.1747	TOTAL	−27,540.6	46.1238	4.6124
Differences (Complex)							
Energy Component	Average	Std. Dev.	Err. of Mean	Energy Component	Average	Std. Dev.	Err. of Mean
VDWAALS	−239.588	8.5386	0.8539	VDWAALS	−239.588	8.5386	0.8539
EEL	741.5124	66.0836	6.6084	EEL	741.5124	66.0836	6.6084
EGB	−501.319	60.9302	6.093	EPB	−515.362	57.6386	5.7639
ESURF	−30.0511	1.1245	0.1125	ENPOLAR	−28.8851	0.877	0.0877
DELTA G gas	521.9245	66.2058	6.6206	DELTA G gas	521.9245	66.2058	6.6206
DELTA G solv	−531.37	60.2798	6.028	DELTA G solv	−544.247	57.0585	5.7058
DELTA TOTAL	−29.4452	9.8089	0.9809	DELTA TOTAL	−42.3229	18.834	1.8834

MM/GBSA (molecular mechanics generalized Born surface area), MM/PBSA (molecular mechanics Poisson–Boltzmann surface area), VDWAALS (van der Waals), EEL (electrostatic), EGB (polar solvation energy of MM-GBSA), ESURF (nonpolar solvation energy), Delta G gas (net gas phase energy), Delta G solv (net solvation energy), Delta Total (net energy of system).

## Data Availability

The data presented in this study are available within the article.

## Abbreviations

AMBER	Assisted Model Building with Energy Refinement tool
BPGA	Bacterial pan-genome analysis tool
CAI	Codon adaptation index
CD-HIT	Cluster database at high identity with tolerance
C-Immsimm	Computational immune simulation
CTL	Cytotoxic T lymphocytes
GRAVY	Grand average of hydropathicity
H.B	Hydrogen bonding
HTL	Helper T lymphocytes
IEDB	Immune epitope database resource
IFNg	Interferon gamma
IRMSD	Interface root-mean-square deviation
JCAT	Java codon adaptation tool
MD	Molecular dynamics
MEPTWV	Multi-epitope peptide *T. whipplei* vaccine
MMGBSA	Molecular mechanics generalized born surface area
MMPBSA	Molecular mechanics Poisson–Boltzmann surface area
MW	Molecular weight
NCBI	National center of biotechnology information
PCR	Polymerase chain reaction
PDBsum	Protein databank structural summaries
PSORTB	Protein subcellular localization prediction tool
RMSD	Root-mean-square deviation
RMSF	Root-mean-square fluctuations
TLR4	Toll-like receptor 4
TMHMM	Transmembrane Helices; Hidden Markov Model
